# An Improved Unscented Particle Filter Approach for Multi-Sensor Fusion Target Tracking

**DOI:** 10.3390/s20236842

**Published:** 2020-11-30

**Authors:** Junhai Luo, Zhiyan Wang, Yanping Chen, Man Wu, Yang Yang

**Affiliations:** School of Information and Communication Engineering, University of Electronic Science and Technology of China, Chengdu 611731, China; 201922011615@std.uestc.edu.cn (Z.W.); 201822011421@std.uestc.edu.cn (Y.C.); 201821011420@std.uestc.edu.cn (M.W.); 201822011422@std.uestc.edu.cn (Y.Y.)

**Keywords:** multi-sensor fusion, target tracking, improved unscented particle filter, data fusion

## Abstract

In this paper, a new approach of multi-sensor fusion algorithm based on the improved unscented particle filter (IUPF) and a new multi-sensor distributed fusion model are proposed. Additionally, we employ a novel multi-target tracking algorithm that combines the joint probabilistic data association (JPDA) algorithm and the IUPF algorithm. To improve the real-time performance of the UPF algorithm for the maneuvering target, minimum skew simplex unscented transform combined with a scaled unscented transform is utilized, which significantly reduces the calculation of UPF sample selection. Moreover, a self-adaptive gain modification coefficient is defined to solve the low accuracy problem caused by the sigma point reduction, and the problem of particle degradation is solved by modifying the weights calculation method. In addition, a new multi-sensor fusion model is proposed, which better integrates radar and infrared sensors. Simulation results show that IUPF effectively improves real-time performance while ensuring the tracking accuracy compared with other algorithms. Besides, compared with the traditional distributed fusion architecture, the proposed new architecture makes better use of the advantages of radar and an infrared sensor and improves the tracking accuracy.

## 1. Introduction

Multi-sensor fusion maneuvering target tracking is one of the subjects that has been investigated during the last decades [1,2]. Multiple sensors provide multi-source information such as distance, angel, audio, and image compared with a single sensor. The more target information, the more accurate the state estimation, so multi-sensor fusion has better performance. Multi-sensor data fusion technology in the low-altitude airspace integrates data from different information sources to achieve accurate estimation of targets and real-time assessment of airspace conditions, and is convenient for anti-aircraft research in the meantime.

Radar, with a longer detection range and accurate distance information, captures the target’s distance, angle, and other information by transmitting electromagnetic radiation; however, it is vulnerable to anti-radiation missiles and electronic interference and a low-level bind spot exists as well [3]. In contrast to the mentioned disadvantages of radar, the infrared sensor has powerful anti-jamming capability, higher angle measuring precision, and better target recognizing ability without radiating any energy. However, it cannot directly obtain the distance information of target [4]. Thus, comprehensive use of the advantages of these two sensors can not only achieve better target recognition and tracking, but also lays the foundation for subsequent anti-UAV hard destruction. Based on the advantages and disadvantages of the radar and infrared sensors mentioned above, many scholars fuse radar and infrared sensors for target tracking [5,6], but the literature [7] proposes that the traditional distributed fusion architecture cannot make better use of the advantages of the two.

Filtering estimation is the main problem in multi-sensor fusion target tracking. Kalman filter (KF) [8] can be used in general nonlinear state estimation problems, but filtering divergence will occur under the strong nonlinear and non-Gaussian conditions, which causes lower tracking accuracy [9]. Unscented Kalman filter (UKF) [10] is easier to implement and more accurate than the Kalman filter. Although the particle filter (PF) [11,12] has shown better performance for dealing with parameter estimation and state filtering of non-Gaussian nonlinear time-varying systems, there are still two weaknesses: a large number of samples are used to approximate the posterior probability density of the system and the problem of particle degradation [13]. Some proposed methods have improvements such as combining extended Kalman filter with a particle filter (EKF-PF) [14] and combining unscented Kalman filter with particle filtering (UPF) [15]. In UPF, using UKF to obtain the importance density function instead of the importance density function in the classic PF algorithm, the tracking accuracy can be improved. Compared with PF and UKF, UPF undoubtedly has higher tracking accuracy [16], but two shortcomings cannot be ignored: one is that the calculation takes a long time as a lot of so-called sigma points are chosen, which cannot meet the tracking real-time requirements of any systems [17]. The other is that the loss of validity of importance sampling leads to a more serious problem of particle degradation.

Aiming at the above problem, this paper proposes an improved UPF (IUPF) approach based on a novel fusion architecture for multi-sensor fusion target tracking and applies it to the multi-target tracking by combining with joint probabilistic data association (JPDA) algorithm [18]. The main contributions of this paper are as follows.

- Aiming at the problem that the traditional distributed fusion architecture cannot make better use of the advantages of radar and infrared sensors, this paper proposes an improved distributed fusion architecture, which effectively utilizes the advantages of radar and infrared sensors, thus improving tracking performance and avoiding tracking divergence.
- An adaptive tracking algorithm with good tracking accuracy, great real-time performance, and strong robustness is proposed. Although the traditional UPF method has high tracking accuracy, real-time performance is low due to the high computational complexity. Different from UPF, the IUPF can improve general calculation speed and tracking accuracy. The minimal skew simplex unscented transform (MSSUT) [19] and scaled unscented transform (SUT) [20] are utilized to effectively reduce the amount of calculation of sample selection, and a self-adaptive gain modification coefficient is defined to solve the low-accuracy problem caused by the sigma point reduction in this paper. By rewriting the formula for calculating the weight of importance, the problem of particle degradation can be solved.
- Applying IUPF to multi-target tracking, the proposed method is a simple JPDA algorithm with IUPF, which can deal with non-trivial nonlinear conditions and improve the accuracy.

This paper is organized as follows. In Section 2, the corresponding theoretical support is introduced. Section 3 and Section 4 provide the details of the proposed IUPF algorithm, improved fusion model, and multi-target tracking algorithm. Two simulation scenarios are used to evaluate the proposed approaches in Section 5, and the simulation results analysis is shown in Section 6. Finally, the conclusion and future outlook are discussed in Section 7.

## 2. Related Work and Problem Statement

In this section, we will discuss the excellent work related to UPF and JPDA in recent years, and also introduce the target motion model model and sensor observation model.

### 2.1. Related Work

The authors of [21] first proposed the UPF algorithm in 2001. They used a bank of unscented filters to obtain the importance proposal distribution based on sequential importance sampling, which made efficient use of the latest available information. The results show that the algorithm strongly outperforms standard particle filtering and other nonlinear filtering methods. Since UPF was created, there have been many works based on UPF. Unfortunately, a number of works have applied UPF to predict lithium-ion battery remaining useful life [22,23], while there are few works on target tracking. The main reason is the poor real-time performance of UPF due to high computational cost. It was not until Julier proposed the minimal skew simplex unscented transform and the spherical simplex unscented transform that the computational complexity of UPF was reduced. In order to achieve the high precision of celestial navigation in the deep space environment, the authors of [24] proposed the spherical simplex unscented particle filter (SSUPF) algorithm. They used the spherical simplex unscented transform instead of UT, then used spherical simplex unscented Kalman filter (SSUKF) to generate the importance density function of the particle filter. Based on the simulation results, they concluded that the SSUPF method can greatly improve the performance of the navigation system compared with the UKF, SSUKF, and unscented PF (UPF). However, the algorithm only reduces the computational complexity by 24%. The authors of [25] proposed a scale-corrected minimal skew simplex sampling unscented Kalman filter (UKF) algorithm for the permanent magnet (PM) brushless DC motors (BLDCM) sensorless control. They used the MSSUT to reduce the amount of calculation and used SUT to overcome local effects. The simulation results showed that this method reduces the total time from 3.43 s to 2.51 s.

In addition, the authors of [26] pointed out that UPF prior distribution calculation may cause numerical sensitivity problems, which in turn causes particle degradation, and performance can be improved by solving numerically sensitive problems.

The above references are based on single-target tracking or positioning. To the best of our knowledge, there is no work that applies the UPF algorithm to multi-target tracking up to now. The main reason for this is that the traditional IUPF algorithm has lower real-time performance and multi-target tracking is better complex than single-target tracking. We apply the IUPF algorithm to multi-target tracking as the IUPF algorithm proposed in this paper greatly reduces the computational complexity. For multi-target tracking, the key issue is how to get the associations between measurements and targets (i.e., data association). JPDA is a classical data association algorithm that has superior performance in the background of strong clutter and moderate computational cost. Therefore, the JPDA algorithm is a better data association algorithm in the field of multi-target tracking. Some researchers combine JPDA with other target tracking algorithms for multi-target tracking, and the results show that this method has better filter accuracy than using the JPDA algorithm alone. In [27], an improved probabilistic data association (IPDA) was used for the multi-target association, then a mixed Kalman/H∞ filtering fusing the covariance of state estimation was proposed for the fast and robust state estimation of video targets. In [28], the suboptimal JPDA technology was used to achieve data association for multi-target tracking, and then a Gaussian particle filter was used for target tracking, which could deal with non-trivial nonlinear conditions and improve the accuracy of multi-target tracking. In [29], to accommodate the nonlinear measurements, the well-known UKF was utilized in the local JPDA filter.

In summary, the UPF tracking accuracy is higher than other tracking methods. The main problems are that numerical sensitivity leads to particle degradation and low real-time performance. In addition, there are few works on how to reduce the computational complexity of UPF. We will solve the above problems in this article and also study the application of IUPF and JPDA to multi-target tracking.

### 2.2. Target Motion Model

We assume that the maneuvering target dynamics are modeled in Cartesian coordinates and moves on the 2-D plane. Then, the target state vector $X_{k}$ at time *k* is denoted as

(1) $$X_{k}=F_{k-1}X_{k-1}+G_{k-1}v_{k-1}$$

where $X_{k}={\left[x_{k},{\dot{x}}_{k},y_{k},{\dot{y}}_{k}\right]}^{T}$, (*x, y*) is the position coordinates along *x*, *y* axes, respectively, and $\left(\dot{x},\dot{y}\right)$ is the velocity vector. *F* is state transition matrix, *G* is the noise transition matrix, and *v* is a zero-mean white Gaussian noise sequence with known variance $Q_{k-1}$.

In this paper, the system motion model is built by nearly constant velocity model (NCV) and nearly constant turn (NCT), and their *F* and *G* matrix is described as

$$\left\{ \begin{array}{l} F_{CV}=\left[\begin{array}{cccc} 1 & T & 0 & 0\\ 0 & 1 & 0 & 0\\ 0 & 0 & 1 & T\\ 0 & 0 & 0 & 1 \end{array}\right],G_{CV}=\left[\begin{array}{cc} \frac{T^{2}}{2} & 0\\ T & 0\\ 0 & \frac{T^{2}}{2}\\ 0 & T \end{array}\right]\\ F_{CT}=\left[\begin{array}{cccc} 1 & \frac{sin\left(\omega T\right)}{\omega } & 0 & \frac{cos\left(\omega T\right)-1}{\omega }\\ 0 & cos\left(\omega T\right) & 0 & -sin\left(\omega T\right)\\ 0 & \frac{1-cos\left(\omega T\right)}{\omega } & 1 & \frac{sin\left(\omega T\right)}{\omega }\\ 0 & sin\left(\omega T\right) & 0 & cos\left(\omega T\right) \end{array}\right]\\ G_{CT}=\left[\begin{array}{cc} \frac{T^{2}}{2} & 0\\ T & 0\\ 0 & \frac{T^{2}}{2}\\ 0 & T \end{array}\right] \end{array}\right.$$

where *T* is the sampling period of the sensor and ω is the rotation rate.

### 2.3. Radar and Infrared Sensor Observation Models

For radar, the target-generated range and bearing measurements are modeled by

(2) $$Z_{k}^{ra}=\left[\begin{array}{c} r_{k}^{ra}\\ \vartheta_{k}^{ra} \end{array}\right]+w_{k}^{ra}$$

For infrared sensors, the target bearing measurement is modeled by

(3) $$Z_{k}^{in}=\left[\vartheta_{k}^{in}\right]+w_{k}^{in}$$

where $r_{k}^{ra}=\sqrt{{\left(x_{k}-x_{s,k}^{ra}\right)}^{2}+{\left(y_{k}-y_{s,k}^{ra}\right)}^{2}}$, which is a target-generated range measurement; $\left(x_{k},y_{k}\right)$ is target position; and $\left(x_{s,k}^{ra},y_{s,k}^{ra}\right)$ is radar position. $w_{k}$ is a sample of zero-mean white Gaussian noise sequence with known variance $R_{k}$. $\vartheta_{k}$ is the target bearing measurement and can be calculated by

(4) $$\vartheta_{k}=\{\begin{array}{ll} \arctan(d_{y}/d_{x}), & for\;d_{x}>0,\;d_{y}>0\\ \;\pi +\arctan(d_{y}/d_{x}), & \;for\;d_{y}<0\\ 2\pi +\arctan(d_{y}/d_{x}), & for\;d_{x}<0,\;d_{y}>0\\ \pi /2, & for\;d_{x}>0,\;d_{y}=0\\ 3\pi /2, & for\;d_{x}<0,\;d_{y}=0\\ undefined, & for\;d_{x}=0,\;d_{y}=0 \end{array}$$

where $d_{x}=x_{k}-x_{s,k}$, $d_{y}=y_{k}-y_{s,k}$, and $\left(x_{s,k},y_{s,k}\right)$ is sensor position.

## 3. The IUPF Algorithm

In this section, we propose the IUPF algorithm. Compared with other tracking methods, IUPF has three advantages: first, it can deal with any nonlinear or non-Gaussian problems in non-dynamics due to being designed based on particle filters; second, it can effectively utilize the latest available information; third, it has good real-time accuracy and strong robustness.

The traditional UPF algorithm obtains the importance proposal distribution through the UKF method, thereby obtaining a posterior probability distribution with a better fit. However, an unexpected result is that 2*n* + 1 sigma points selected will result in lower efficiency, especially when combined with other algorithms for maneuvering target tracking. Besides, the importance of sampling may be invalid due to the numerical sensitivity problem, and finally the particle degradation problem will occur. To solve the above problems, this paper proposes the IUPF algorithm. In IUPF, by using the minimum skew simplex sigma point sampling instead of the traditional symmetric unaccented sigma point sampling, the sigma points can be reduced by 50%, so the calculation complexity of the algorithm is reduced from O[(2n+1)N] to O[(n+2)N]. Furthermore, scaled unscented transform parameters are introduced to remove errors caused by non-local effects of the high-dimensional state space under non-Gaussian conditions. Furthermore, a self-adaptive gain modification coefficient is defined to solve the low-accuracy problem caused by the sigma point reduction. Finally, we rewrite the corresponding weight calculation formula, which can solve the problem of particle degradation. The specific algorithm is as follows.

**For k = 0**

Initialization: Draw *N* particles ${\left\{X_{0}^{\left(i\right)}\right\}}_{i=1}^{N}$ from the prior distribution $p\left(X_{0}\right)\sim N\left(X_{0}^{\left(i\right)};{\hat{X}}_{0},P_{0}\right)$ and set the initial weight of each particle as $w_{0}^{\left(i\right)}=\frac{1}{N}$.

**For k = 1, 2, …**

**(1) Importance sampling**

For *i* = 1, 2, …, *N*, Update the state for each particle using the UKF.

**Step 1.** Use minimal skew simplex UT to choose *n* + 1 sigma points rather than UT to choose 2*n* + 1 sigma points. Thus, two associated weights for every sigma point are changed compared to UPF. Using the weight ratio correction method, we can get

(5) $$\{\begin{array}{l} W_{m_{0}}^{\left(i\right)}=\frac{\lambda }{n+\lambda }\\ W_{c_{0}}^{\left(i\right)}=\frac{\lambda }{\left(n+\lambda \right)\left(1-\alpha^{2}+\beta \right)} \end{array}$$

(6) $$\left\{ \begin{array}{l} W_{0}^{m,\left(i\right)}=\frac{W_{m0}^{\left(i\right)}}{\alpha^{2}}+\left(1-\frac{1}{\alpha^{2}}\right)\\ W_{0}^{c,\left(i\right)}=\frac{W_{c0}^{\left(i\right)}}{\alpha^{2}}+\left(1-\alpha^{2}+\beta \right)\\ W_{1}=\frac{1}{\alpha^{2}\cdot 2^{n}}\left(1-W_{0}^{m,\left(i\right)}\right),j=1\\ W_{2}=W_{1},j=2\\ W_{j}^{m,\left(i\right)}=W_{j}^{c,\left(i\right)}=\frac{2^{j-1}}{\alpha^{2}}W_{1},j=3,4,\ldots ,n+1 \end{array}\right.$$

where $W_{c0}$ and $W_{m0}$ are the initial weights for the range [0,1]. $W_{i}^{m}$ and $W_{i}^{c}$ are the weights calculated by the weight ratio correction method. $\lambda =\alpha^{2}\left(n+\kappa \right)-n$ is the scaling parameter and κ is a candidate coefficient to ensure that $\left(n+\lambda \right)P_{k}$ is a positive semi-definite matrix. $W_{i}^{m}$ is used when computing the mean and $W_{i}^{c}$ is used when recovering the covariance of the Gaussian. α is a scale factor that controls the distribution of sigma points for the range are $\left[10^{-4},1\right]$ and β introduces the prior distribution information of random variables.

According to the minimum skew simplex UT, the 1-dimensional minimum skew simplex UT for a single line of sigma points is

$$\xi_{0}^{1,\left(i\right)}=\left[0\right],\xi_{1}^{1,\left(i\right)}=\left[\frac{-1}{\sqrt{2W_{1}}}\right],\xi_{2}^{1,\left(i\right)}=\left[\frac{1}{\sqrt{2W_{1}}}\right]$$

then the *l* (*l* = 2, 3, ..., *n*) -dimensional expansion vector sigma points can be depicted as

$$\xi_{j,l}^{\left(i\right)}=\{\begin{array}{l} {\left[\xi_{0,l-1}^{\left(i\right)},0\right]}^{T},j=0\\ {\left[\xi_{j,l}^{\left(i\right)},\frac{-1}{\sqrt{2W_{j}}}\right]}^{T},j=1,2,\ldots ,l\\ {\left[0^{l},\frac{1}{\sqrt{2W_{j}}}\right]}^{T},j=l+1 \end{array}$$

where $\xi_{j,l}^{i}$ is the *i*th sigma point of the *l*-dimensional random variable, and $\xi_{j}=\left(\xi_{j}^{1},\xi_{j}^{2},\ldots ,\xi_{j}^{n}\right)$. Therefore, the sampling point at time *k −* 1 is

(7) $$\xi_{j,k}^{\left(i\right)}={\left[{\hat{X}}_{k},{\hat{X}}_{k}+\sqrt{\alpha^{2}\left(n+\lambda \right)}\xi_{1}^{\left(i\right)},\ldots ,{\hat{X}}_{k}+\sqrt{\alpha^{2}\left(n+\lambda \right)}\xi_{n}^{\left(i\right)}\right]}^{T}$$

**Step 2**. State prediction. Equation (1) is used to calculate the result of a given sigma point propagating; then, we can obtain the state mean and covariance of the random variable:

(8) $$\{\begin{array}{l} {\hat{X}}_{k|k-1}^{\left(i\right)}=\sum_{j=0}^{n}W_{j}^{m,\left(i\right)}\xi_{j,k|k-1}^{\left(i\right)}\\ P_{k|k-1}^{\left(i\right)}=\sum_{j=0}^{n}W_{i}^{c,\left(i\right)}\left(\xi_{j,k|k-1}^{\left(i\right)}-{\hat{X}}_{k|k-1}^{\left(i\right)}\right){\left(\xi_{j,k|k-1}^{\left(i\right)}-{\hat{X}}_{k|k-1}^{\left(i\right)}\right)}^{T} \end{array}$$

**Step 3.** Measurement prediction. The calculation result of each sigma point propagated $Z_{i,k|k-1}$ is calculated by (2) and (3), and then the further predicted values of the measured mean and covariance are calculated by

(9) $$\left\{ \begin{array}{l} {\hat{Z}}_{k|k-1}^{\left(i\right)}=\sum_{i=0}^{n}W_{j}^{m,\left(i\right)}Z_{j,k|k-1}^{\left(i\right)}\\ P_{Z_{K}}^{\left(i\right)}=\alpha^{2}\sum_{j=0}^{n}W_{j}^{c,\left(i\right)}\left(Z_{j,k|k-1}^{\left(i\right)}-{\hat{Z}}_{k|k-1}^{\left(i\right)}\right){\left(Z_{j,k|k-1}^{\left(i\right)}-{\hat{Z}}_{k|k-1}^{\left(i\right)}\right)}^{T}\\ P_{Xz}^{\left(i\right)}=\sum_{j=0}^{n}W_{j}^{c,\left(i\right)}\left(x_{j,k|k-1}^{\left(i\right)}-{\hat{x}}_{k|k-1}^{\left(i\right)}\right){\left(Z_{i,k|k-1}-{\hat{Z}}_{k|k-1}\right)}^{T} \end{array}\right.$$

where ${\hat{Z}}_{k|k-1}^{\left(i\right)}$ and $P_{z_{k}}^{\left(i\right)}$, respectively, are the measured predicted and its covariance, and $P_{Xz}^{\left(i\right)}$ is the covariance between the predicted state and the measured predicted value.

**Step 4.** Filter updating. After obtaining a new measurement, based on the predicted value gained in Step 3, we can calculate the Kalman gain matrix $K_{k}$, then we update the state estimate ${\hat{X}}_{k}^{\left(i\right)}$ and its covariance $P_{k}^{\left(i\right)}$.

(10) $$\{\begin{array}{l} K_{k}=P_{xz}^{\left(i\right)}{\left(P_{xz}^{\left(i\right)}\right)}^{-1}\\ {\hat{X}}_{k}^{\left(i\right)}={\hat{X}}_{k|k-1}^{\left(i\right)}+K_{k}\left(Z_{k}-{\hat{Z}}_{k|k-1}^{\left(i\right)}\right)\\ P_{k}^{\left(i\right)}=P_{k|k-1}^{\left(i\right)}-K_{k}P_{Z_{k}}^{\left(i\right)}{\left(K_{k}\right)}^{T} \end{array}$$

**Step 5.** Update importance weight. Sampling ${\left\{X_{k}^{\left(i\right)}\right\}}_{i=1}^{N}$ from the state probability density

(11) $${\left\{X_{k}^{\left(i\right)}\right\}}_{i=1}^{N}\sim q\left(X_{k}^{\left(i\right)}|X_{k-1}^{\left(i\right)}{}_{,}Z_{k}\right)=N\left(X_{k}^{\left(i\right)};{\hat{X}}_{k}^{\left(i\right)},P_{k}^{\left(i\right)}\right)$$

In the traditional UPF, importance weight is calculated by

(12) $$w_{k}^{\left(i\right)}\propto w_{k-1}^{\left(i\right)}\frac{p\left(Z_{k}|X_{k}^{\left(i\right)}\right)p\left(X_{k}^{\left(i\right)}|X_{k}^{\left(i-1\right)}\right)}{q\left(X_{k}^{\left(i\right)}|X_{k-1}^{\left(i\right)},Z_{k}\right)}$$

We assume ${\left\{X_{k-1}^{\left(i\right)}\right\}}_{i=1}^{N}\sim N\left(X_{k-1}^{\left(i\right)};{\hat{X}}_{k-1}^{\left(i\right)},P_{k-1}^{\left(i\right)}\right)$ at time *k* – 1, so the state distribution is $p\left(X_{k}^{\left(i\right)}|X_{k-1}^{\left(i\right)}\right)=N\left(X_{k}-F\left({\hat{X}}_{k-1}^{\left(i\right)}\right);0,Q_{k}\right)$, and $Q_{k}=diag\left({\sigma_{1}}^{2},{\sigma_{2}}^{2},\ldots ,{\sigma_{n}}^{2}\right)$. The state prediction error $e_{k}=X_{k}-F\left({\hat{X}}_{k-1}\right)$ should be within the range of the state space $Q_{k}$ includes. However, because of the large value of $P_{k}$, the larger sampling uncertainty is introduced, which means that $d_{k}^{i}=\|X_{k}^{i}-F\left({\hat{X}}_{k-1}^{\left(i\right)})\right\|$ and $\sigma max{\left\{{\sigma_{1}}^{2},{\sigma_{2}}^{1},\ldots ,{\sigma_{n}}^{2}\right\}}_{max}$ are not on the same order of magnitude. In general, the process noise is relatively small and the state estimation error may be relatively large, which will cause numerical sensitivity during calculation and further cause the problem of particle degradation. According to the above analysis, we can find that the root of the problem is the prior distribution $p\left(X_{k}^{\left(i\right)}|X_{k-1}^{\left(i\right)}\right)$. To solve this problem, we use the pdf $p\left(X_{k}^{\left(i\right)}|Z_{k-1}\right)$ instead of the pdf $p\left(X_{k}^{\left(i\right)}|X_{k-1}^{\left(i\right)}\right)$, so we have

(13) $$W_{k}^{\left(i\right)}\propto W_{k-1}^{\left(i\right)}\frac{p\left(Z_{k}|X_{k}^{\left(i\right)}\right)p\left(X_{k}^{\left(i\right)}|Z_{k-1}\right)}{q\left(X_{k}^{\left(i\right)}|X_{k-1}^{\left(i\right)},Z_{k}\right)}$$

where $p\left(Z_{k}|X_{k}^{\left(i\right)}\right)\sim N\left(Z_{i}-h\left({\hat{X}}_{k}^{\left(i\right)}\right);0,R_{k}\right)$, $p\left(X_{k}^{\left(i\right)}|Z_{k-1}\right)\sim N\left(X_{k}^{\left(i\right)};{\hat{X}}_{k|k-1}^{\left(i\right)},P_{k|k-1}^{\left(i\right)}\right)$.

Compared with (12), Equation (13) is calculated by predictive distribution instead of the prior method. As the predictive and filtering covariances are comparable in magnitude, the numerical sensitivity problem will be solved and the distribution character of the weights is improved. Therefore, the particle degradation due to calculation overflow also will effectively avoided.

Then, we estimate the weight of each particle and normalize it:

(14) $${\tilde{W}}_{k}^{\left(i\right)}=\frac{w_{k}^{\left(i\right)}}{\sum_{i=1}^{N}w_{k}^{\left(i\right)}}$$

**(2) Self-adaptive gain modification**

The particle predicted residual is derived from the measurement and one-step predicted state:

(15) $$\varepsilon_{k}^{\left(i\right)}=Z_{k}-{\hat{Z}}_{k|k-1}^{\left(i\right)}$$

The corresponding covariance is

$$S_{\varepsilon }=\alpha^{2}\overset{n}{\underset{j=0}{\sum }}W_{j}^{c,\left(i\right)}\left(Z_{j,k|k-1}^{\left(i\right)}-{\hat{Z}}_{k|k-1}^{\left(i\right)}\right){\left(Z_{j,k|k-1}^{\left(i\right)}-{\hat{Z}}_{k|k-1}^{\left(i\right)}\right)}^{T}$$

According to (11) and (13), the importance weight is determined by ${\hat{X}}_{k}^{\left(i\right)}$,$\;P_{k}^{\left(i\right)}$ to a certain extent. From (10), it can be seen that ${\hat{X}}_{k}^{\left(i\right)}$,$\;P_{k}^{\left(i\right)}$ is related to gain. When a moving target suddenly accelerates or turns or decelerates to a stop, ${\hat{X}}_{k-1}^{\left(i\right)}$ will be more inaccurate, so we need to adjust gain in (10) to better balance the contribution of sensor measurement and ${\hat{X}}_{k-1}^{\left(i\right)}$. From (9), we can see that gain and $P_{k}^{\left(i\right)}$ is related to predicted residual. Therefore, we can use the predicted residuals to adjust the state and measured covariance in real time to adjust the gain and predicted covariance. In turn, the gain will adjust the particle importance weight.

We define a new kind of normalized statistics:

(16) $$\tilde{\Delta }\varepsilon_{k}=\frac{tr\left(\varepsilon_{k}^{\left(i\right)}{\left(\varepsilon_{k}^{\left(i\right)}\right)}^{T}\right)-tr\left(S_{\varepsilon }\right)}{tr\left(\varepsilon_{k}^{\left(i\right)}{\left(\varepsilon_{k}^{\left(i\right)}\right)}^{T}\right)}$$

where tr(**A**) denotes the trace of matrix **A.**

Then, based on the adaptive control method [30,31], we can obtain an adaptive factor:

(17) $$\theta_{k}^{\left(i\right)}=\{\begin{array}{l} 1,tr\left(\varepsilon_{k}^{\left(i\right)}{\left(\varepsilon_{k}^{\left(i\right)}\right)}^{T}\right)\leq tr\left(S_{\varepsilon }\right);\\ \frac{tr\left(\varepsilon_{k}^{\left(i\right)}{\left(\varepsilon_{k}^{\left(i\right)}\right)}^{T}\right)-tr\left(S_{\varepsilon }\right)}{tr\left(\varepsilon_{k}^{\left(i\right)}{\left(\varepsilon_{k}^{\left(i\right)}\right)}^{T}\right)},tr\left(\varepsilon_{k,i}{\left(\varepsilon_{k,i}\right)}^{T}\right)>tr\left(S_{\varepsilon }\right) \end{array}$$

The adaptive factor can adjust gain and covariance through

(18) $$\left\{ \begin{array}{c} P_{Z_{k}}^{\left(i\right)}=\alpha^{2}\sum_{j=0}^{n}\frac{W_{j}^{c,\left(i\right)}}{\vartheta_{k}^{\left(i\right)}}\left(Z_{j,k|k-1}^{\left(i\right)}-{\hat{Z}}_{k|k-1}^{\left(i\right)}\right){\left(Z_{j,k|k-1}^{\left(i\right)}-{\hat{Z}}_{k|k-1}^{\left(i\right)}\right)}^{T}\\ P_{Xz}^{\left(i\right)}=\sum_{j=0}^{n}\frac{W_{j}^{c,\left(i\right)}}{\vartheta_{k}^{\left(i\right)}}\left(x_{j,k|k-1}^{\left(i\right)}-{\hat{x}}_{k|k-1}^{\left(i\right)}\right){\left(Z_{i,k|k-1}-{\hat{Z}}_{k|k-1}\right)}^{T} \end{array}\right.$$

(19) $$\{\begin{array}{l} K_{k}=P_{xz}^{\left(i\right)}{\left(P_{xz}^{\left(i\right)}\right)}^{-1}\\ P_{k}^{\left(i\right)}=\frac{1}{\vartheta_{k}^{\left(i\right)}}P_{k|k-1}^{\left(i\right)}-K_{k}P_{Z_{k}}^{\left(i\right)}{\left(K_{k}\right)}^{T} \end{array}$$

Submitting (18) and (19) into (10), we derive the following cases.

**Case 0:** If the target is moving steadily, the system model is accurate at present. $\varepsilon_{k}^{\left(i\right)}$ will remain stable and $\theta_{k}^{\left(i\right)}$=1. Gain will not be adjusted.

**Case 1:** If the maneuvering target suddenly accelerates or turns, $\varepsilon_{k}^{\left(i\right)}$ will increase and 0 < $\theta_{k}^{\left(i\right)}<1$ for the equation of state cannot predict accurately. Then, the gain in (10) will be larger, which will make particle samples more reasonable at dealing with useful information, and decrease the contribution of ${\hat{X}}_{k-1}^{\left(i\right)}$ and increase the contribution of $Z_{k}$. Therefore, we can get a proposal distribution that is closer to the posterior distribution for importance sampling process. 

Therefore, introducing a control factor to adaptively adjust the gain according to the prediction residual can reduce the model error in real time and get a better distribution of recommendations, which will greatly improve the filtering accuracy.

**(3) Selective resampling.**

Resampling was performed when the effective sample numbers fell below 75% of the total number of particles rather than after each observation [32]. According to the work in [33], an estimate of *N_eff_* is given by

(20) $$N_{eff}=\frac{1}{\sum_{i=1}^{N}{\left({\tilde{\omega }}_{k}^{\left(i\right)}\right)}^{2}}$$

where *N* is the total number of particles. When *N_eff_* < *N_th_* = 0.75*N*, the systematic resampling (SR) [34] algorithm is used to resample *N* particles from ${\left\{X_{k}^{\left(i\right)},w_{k}^{\left(i\right)}\right\}}_{i=1}^{N}$.

**(4) Output state estimation**

After resampling, ${\left\{X_{k}^{\left(i\right)},w_{k}^{\left(i\right)}\right\}}_{i=1}^{N}$ is changed to ${\left\{X_{k}^{\left(i\right)},\frac{1}{N}\right\}}_{i=1}^{N}$, and the importance weight of every particle is 1/*N*. Then, we can obtain the state estimate

(21) $${\hat{X}}_{k}=\frac{1}{N}\sum_{i=1}^{N}{\hat{X}}_{k}^{\left(i\right)}$$

## 4. Multi-Sensor Fusion Target Tracking Algorithm Based on IUPF

To successfully apply the IUPF algorithm to the field of multi-sensor fusion target tracking, this section proposes a multi-sensor fusion single target and multi-target tracking method based on IUPF. The contents of it mainly include the following.

(1) An improved distributed fusion model of radar and infrared sensors for single target tracking.
(2) A multi-target tracking algorithm based on the JPDA algorithm and IUPF multi-sensor fusion.

### 4.1. Improved Distributed Multi-Sensor Fusion Model 

**Figure 1:**
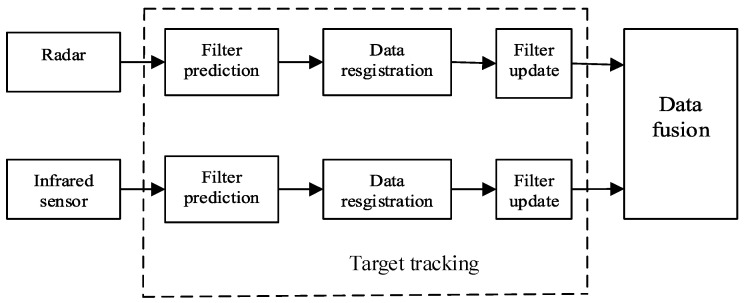
Traditional distributed fusion model for single target tracking.

In the field of multi-sensor fusion, distributed fusion architecture is often used. In distributed fusion, each sensor processes its measurement results to generate local estimates and error covariance, and then sends them to the fusion node to merge them into global state estimation as well as the estimated error covariance [35,36]. Figure 1 shows the traditional distributed fusion model for radar and infrared sensors [37]. For single target tracking, radar and infrared sensors, respectively, track the target and form the relevant target trajectory in their local information processing center, then the local trajectories are sent to the fusion center to perform data fusion. However, for the traditional model, as the infrared sensors can only obtain the angle information of the target and the radar provides the angel and distance information, effectively using the distance information provided by radar to improve the accuracy of target tracking has become a critical problem. An improved fusion model based on radar and infrared sensor are proposed to make better use of the advantages of infrared sensor and radar in this paper. 

**Figure 2:**
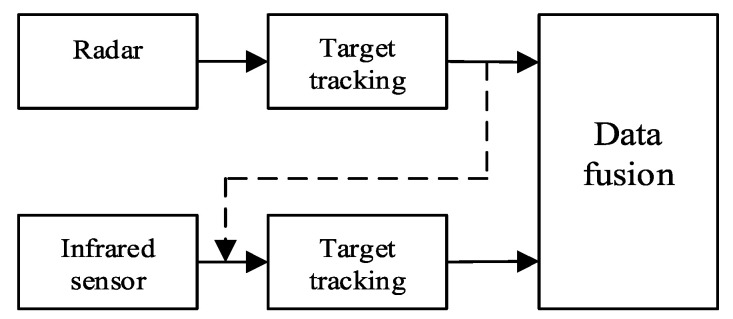
Improved distributed fusion model for single target tracking.

The advantage of the improved model proposed in this paper is that the more accurate long-range target measurement filter result of the radar is used as the status update value of the infrared sensor, and then the target measurement is corrected in time by the infrared sensor observation data with higher accuracy, thereby improving the tracking accuracy of the system. In other words, the improved model can better integrate infrared sensors and radar. Compared with the traditional model, it can reduce the loss of information and improve tracking accuracy. The model is depicted in Figure 2. 

We first perform radar state estimation and update radar observations to obtain radar state estimates and state prediction variances according to the IUPF in Section 3. Then, we use the state vector and covariance gained as the current state update of the value and covariance of the infrared sensor, and update the target of the infrared sensor observations, which is different from the traditional model. Finally, we fuse the estimates of radar and infrared sensors. The specific algorithm is as follows.

**Step 1.** We obtain the measurement value $X_{k|k-1}^{ra}$ and state covariance $P_{k|k-1}^{ra}$ of the radar state at time *k* and calculate the radar filter gain $K_{k}^{ra}$ by (18).

**Step 2.** We calculate the radar status update ${\hat{X}}_{k}^{ra}$ and error covariance $P_{k}^{ra}$ by (10) and (19).

**Step 3.** The value obtained from Step 2 is used as the predicted value and the error covariance of the infrared sensor state at time *k.*

$${\hat{X}}_{k|k-1}^{in}={\hat{X}}_{k}^{ra},\;P_{k|k-1}^{in}=P_{k}^{ra}$$

**Step 4.** We calculate the filter gain of the infrared sensor by (18), predict the state of the measured value of the infrared sensor, and update the filter output by (19).

**Step 5.** We fuse the estimates of radar and infrared sensor in the fusion center, then we obtain the global state estimates and error covariance.

In the fusion center, a hierarchical fusion (HF) algorithm [38] can be utilized to fuse the estimates of radar and infrared sensors. The HF introduces the prior information of each local node, so the global optimal estimate can be obtained, the traffic and calculation amount are moderate compared with other algorithms. 

According to the HF, the fusion equation of the target tracking and the covariance updating equation of the fusion center are

(22) $$P_{k|k}^{-1}{\hat{X}}_{k|k}=P_{k|k-1}^{-1}{\hat{X}}_{k|k-1}+\sum_{l=1}^{N}\left[{\left(P_{k|k}^{l}\right)}^{-1}{\hat{X}}_{k|k}^{l}-{\left(P_{k|k-1}^{l}\right)}^{-1}{\hat{X}}_{k|k-1}^{l}\right]$$

(23) $$P_{k|k}^{-1}=P_{k|k-1}^{-1}+\sum_{l=1}^{N}\left[{\left(P_{k|k}^{l}\right)}^{-1}-{\left(P_{k|k-1}^{l}\right)}^{-1}\right]$$

where ${\hat{X}}_{k|k-1}$, $P_{k|k-1}$ is the overall prior information of the system, and ${\hat{X}}_{k|k-1}^{l}$, $P_{k|k-1}^{l}$, ${\hat{X}}_{k|k}^{l}$, $P_{k|k}^{l}$ is the prior information and estimated values of each local node.

### 4.2. IUPF with JPDA Algorithm for Multi-Target Tracking 

**Figure 3:**
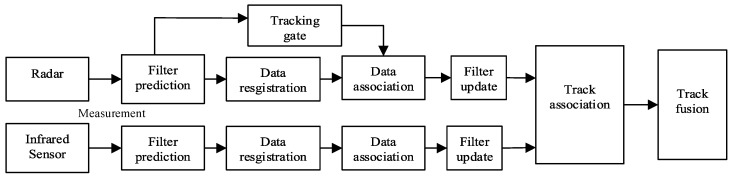
Distributed fusion model for multi-target tracking.

In this section, the JPDA algorithm is embedded in the IUPF algorithm to achieve multi-target tracking, and we assume that we track a fixed number of targets. Although multi-sensor fusion technology for single target tracking has been established, it is not easy to directly extend these algorithms to multi-target tracking. The multi-sensor fusion model for multi-target tracking is shown in Figure 3. In addition to the basic operations of single target tracking, the selection of tracking gates, data association, and track association is required before data fusion as multi-target tracking involves simultaneous tracking of multiple targets.

The tracking gate is to set a threshold to filter the echo measurement. In this paper, an elliptical tracking gate is used as the tracking gate for radar multi-target tracking. Data association is mainly used to associate a verified target with measurement, and then all associated measurements will be used to update the state estimate of the existing object. Besides, in the case of multi-target tracking, the track-to-track association is crucial to find the source from different sensors. However, using only the data association and track association is not enough. We need to combine other excellent tracking methods to improve the accuracy of multi-target tracking. Therefore, we use IUPF to track multi-target, which provides more accurate estimates of state and measurement. The JPDA algorithm is used to solve the measurement-to-track association (i.e., data association) problem. The data association operation is always performed between the state prediction and update of the IUPF process. We assume that there are *m* targets. The specific multi-target tracking algorithm is described as follows.

(1) For *t* = 1, …, *m*, we, respectively, calculate the prediction of the *t*th target state ${\hat{X}}_{k|k-1}^{t}$, covariance $P_{k|k-1}^{t}$, and measurement ${\hat{Z}}_{k|k-1}^{t}$ by (5) to (9) and (18) and (19). Then, we perform space-time registration.
(2) For *t =* 1, …,*m*, we conform the validated measurements (i.e., effective echo within the tracking threshold), and we set *n* = 1, …, *m_k_*, and *m_k_* is the number of the validated measurements. Then, we calculate ${\hat{X}}_{n,k|k-1}^{t,\left(i\right)}$, ${\hat{Z}}_{n,k|k-1}^{t,\left(i\right)}$, $P_{Z_{k}}^{t,(i)}$, $P_{XZ}^{t,(i)}$ by (5) to (9) and (18) and (19).
(3) We use the JPDA algorithm proposed in [18] to receive the association probabilistic of track-to-measurement. The hypothesis-conditioned distribution can be calculated by IUPF instead of Gaussian approximations in the standard JPDA. Then, we can obtain data association probability $\gamma_{n,k}^{t}$.
(4) Filter update. We associate the measurement with the target and then recalculate each target state estimate and its covariance. Equations (10) and (13) are changed as

(24) $$\left\{ \begin{array}{c} K_{k}^{t}=P_{XZ}^{t,(i)}{\left(P_{Z_{k}}^{t,(i)}\right)}^{-1}\\ {\bar{X}}_{k}^{t,\left(i\right)}={\hat{X}}_{k|k-1}^{t,\left(i\right)}+K_{k}^{t}i_{k}^{t}\\ P_{k}^{t,\left(i\right)}=\frac{1}{\vartheta_{k}^{\left(i\right)}}P_{k|k-1}^{t,\left(i\right)}-\left(1-\gamma_{0}^{t,\left(i\right)}\right)K_{k}^{t}+\\ \sum_{n=1}^{m_{k}}\gamma_{n,k}^{t,\left(i\right)}[{\hat{X}}_{n,k}^{t,\left(i\right)}{\left\{{\hat{X}}_{n,k}^{t,\left(i\right)}\right\}}^{T}{\hat{X}}_{k}^{t,\left(i\right)}{\left\{{\hat{X}}_{k}^{t,\left(i\right)}\right\}}^{T}]\\ i_{k}^{t}=\sum_{j=1}^{m_{k}}\gamma_{j,k}^{t}i_{j,k}^{t}\\ i_{n,k}^{t}=Z_{n,k}-Z_{n,k|k-1}^{\left(i\right)} \end{array}\right.$$

where $X_{n,k}^{t,\left(i\right)}$ is the state estimation of the *n*th measurement to the *t*th target at time *k* and $i_{n,k}^{t}$ is the *n*th effective measurement of the *t*th target at time *k*.

(1) For *m* = 1, …, *t*, we can obtain the importance density function $X_{k}^{t,\left(i\right)}∼N\left(X_{k}^{t,\left(i\right)};{\bar{X}}_{k}^{t,\left(i\right)},P_{k}^{t,\left(i\right)}\right)$ of multi-target by (24), then we selective resamples from $X_{k}^{t,\left(i\right)}∼N\left(X_{k}^{t,\left(i\right)};{\bar{X}}_{k}^{t,\left(i\right)},P_{k}^{t,\left(i\right)}\right)$. Then, we compute the state estimation ${\hat{X}}_{k}^{t}$ of the *t*th target at time *k*.
(2) Calculate the state estimation of each sensor through the above steps. Then, use the fuzzy similarity-based correlation algorithm proposed in [39] for track correlation.
(3) Fuse tracks for associated tracks by (22) and (23) to calculate fused state estimation ${\hat{X}}_{k,t}^{F}$.

## 5. Simulation

In this section, the IUPF algorithm is used as a target tracking algorithm, and is used for single-target tracking simulation and multi-target tracking simulation. All these simulations have been done in a PC with a 64-bit Windows 7, Intel core i5, dual-core 2.2 GHZ and 4 GB of RAM. The software used to test the algorithm was MATLAB 2015a.

### 5.1. Single Target Tracking Simulation Experiment

**Figure 4:**
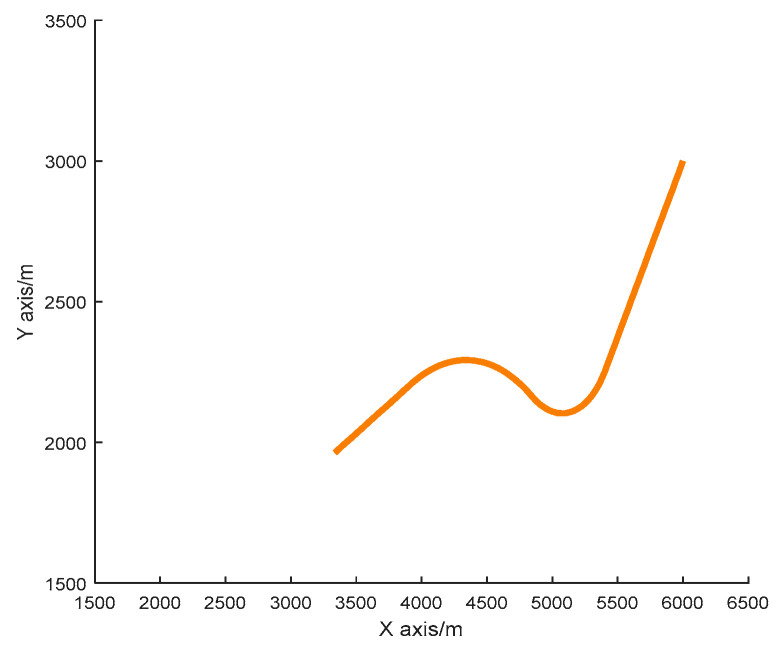
The true trajectory of the single target.

The simulation condition is set as follows. The radar sampling period is 1s, and the infrared sensor sampling period is 0.02 s. The target is moving with an initial state $X_{0}={\left[6000\;m\;,-20\;m/s,\;3000\;m,-25\;m/s\right]}^{T}$, the radar position is [2000 m, 0 m/s, 2000 m, 0 m/s]^*T*^, and the infrared sensor position is [3000 m, 1500 m]^*T*^. Total simulation time is 100 s, and at t = 31s the target starts a fast turn motion with a rotation rate of −0.07 rad/s for the 20 s, then does a slow turn motion with a rotation rate of 0.03 rad/s until the 80s, after that it flies towards the sensor with a constant speed. The radar measurement noise is $\sigma_{r}^{ra}=100\;m$, $\sigma_{\theta }^{ra}=10\;\mathrm{mrad}$. The measurement noise of the infrared sensor is $\sigma_{\theta }^{in}=10\;\mathrm{mrad}$. The basic parameters of the algorithm are α=0.01, β=2. The initial number of particles is 500, and all simulations are done with a 1000 Monte Carlo simulation. The trajectory is shown in Figure 4.

### 5.2. Multi-Target Tracking Simulation Experiment

**Figure 5:**
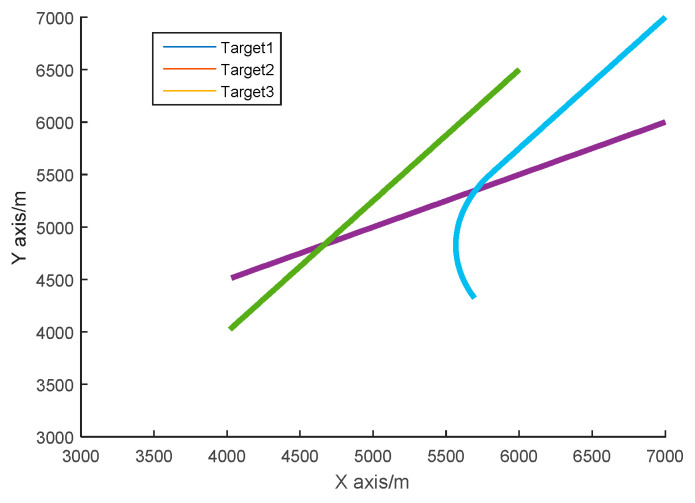
The true trajectories of 3 targets.

The simulation condition is set as follows. The radar sampling period is 1 s, and the infrared sensor sampling period is 0.02 s. The total simulation time is 100 s. In 2D space, three targets are approaching the sensor platform at a uniform speed from a relatively high distance. The radar position is [2000 m, 2000 m]^*T*^, and the infrared sensor position is [3000 m, 1500 m]^*T*^. For the target 1, the initial state is $X_{0}={\left[7000\;m,-30\;m/s,6000\;m,-15\;m/s\right]}^{T}$, and it approaches the sensors at a constant speed. For the target 2, the initial state is $X_{0}={\left[6000\;m,-20\;m/s,6500\;m,-25\right]}^{T}$, and it also approaches the sensors at a constant speed. For target 3, the initial state is $x_{0}={\left[7000\;m,-20\;m/s,7000\;m,-25\;m/s\right]}^{T}$, and it first approaches the sensors with a constant speed until the 60 s, then does a slow turn motion with a rotation rate of 0.03 rad/s. The remaining parameter values are the same as those of the single-target experiment. The trajectories of 3 targets are shown in Figure 5.

## 6. Simulation Results Analysis

The simulation results in this section and the performance of the algorithms described in Section 3 and Section 4 are evaluated. Performance is evaluated by tracker errors, which are given by calculating the difference between the true state and the estimated state. A common indicator of tracking error is the root mean square error (RMSE). In addition, we introduce an average time consumption to compare the real-time performance of IUPFPF, EKF-PF, and UPF. We evaluate it by

$$Average\_time=\frac{1}{N_{c}}\overset{N_{c}}{\underset{j=1}{\sum }}\overset{N_{t}}{\underset{i=1}{\sum }}t_{s}^{i,j}$$

where $t_{s}^{i,j}$ is the time consumption of IUPF in the *i*th sampling period of the *j*th Monte Carlo simulation. Nc represents the number of Monte Carlo simulations and *Nc* = 1000, *Nt* represents the total sampling time of a Monte Carlo simulation and *Nt* = 100 s, and one sampling period is 1 s.

### 6.1. Single Target Tracking Simulation Analysis

In the single-target tracking simulation experiment, we first study the impact of the proposed improved model on the accuracy of target tracking. As the first reference, a traditional distributed fusion structure is used. For a long time, it was a widely used fusion architecture in multi-sensor fusion. As a second reference, a single-sensor radar is used. Then, we verify the performance of the IUPF algorithm based on the improved fusion model. As references, PF, EKF-PF, and UPF were utilized [40]. These algorithms are widely regarded as more accurate methods in filter tracking.

**Figure 6:**
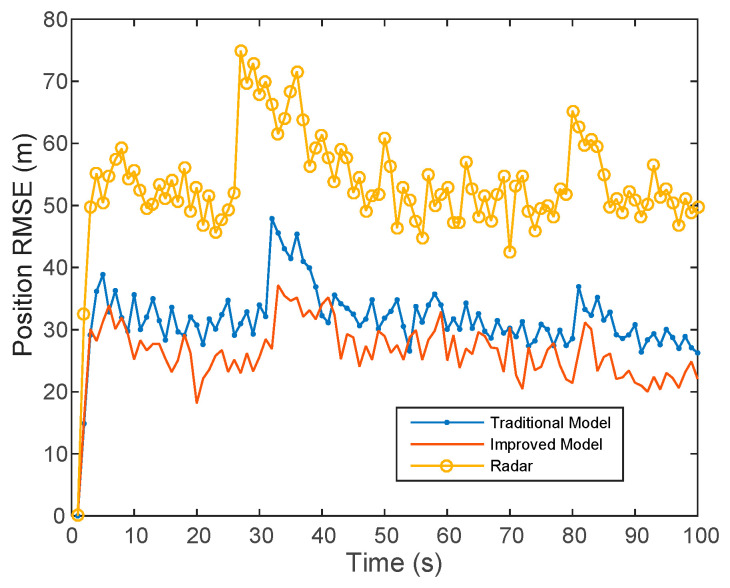
Comparison of fusion models.

From Figure 6 and Table 1, we can find that multi-sensor fusion achieves better optimization and complementation for the fusion of the output results of each sensor compared to a single sensor, which improves the target tracking accuracy significantly. Then, the traditional distributed fusion model is compared with the improved fusion model. The results show that the tracking error of the improved model is smaller than that of the traditional model and more accurate tracking can be achieved.

**Figure 7:**
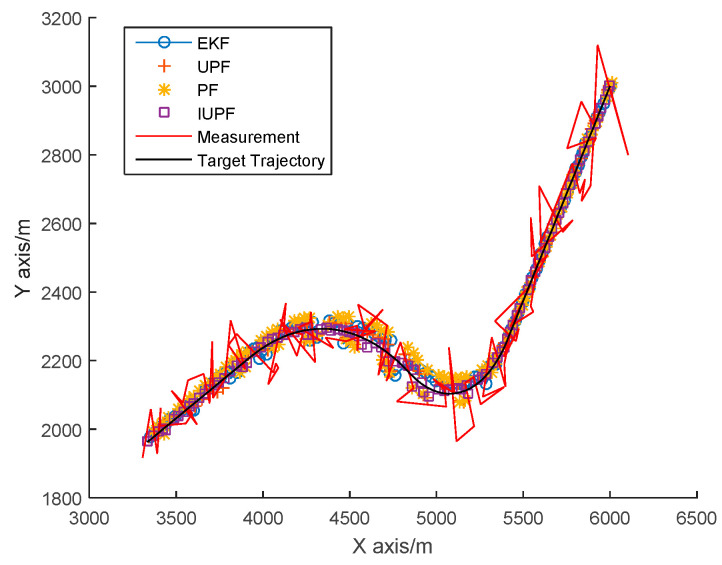
Single-target simulation experiment prediction trajectories.

**Figure 8:**
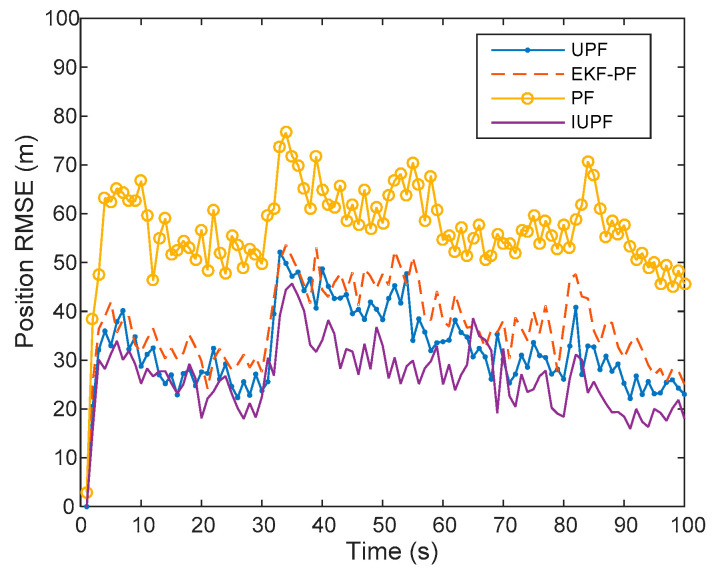
Comparison of position root mean square error (RMSE) in a single-target simulation experiment.

From the predicted trajectories of EKF-PF, PF, UPF, and IUPF in Figure 7, we can see the IUPF is closer to the real trajectory than other algorithms. Figure 8 further compares the position errors of these algorithms. Furthermore, for an easier comparison quantifiable outcomes of the above-mentioned algorithms are compared in Table 2.

From Figure 8 and the average RMSE data in Table 2, it can be seen that the position error of IUPF is smaller than EKF-PF, PF, and UPF. Furthermore, the trajectory error fluctuation of the IUPF algorithm is smaller than EKF-PF, PF, and UPF. We can find that IUPF has a better performance in tracking a maneuvering target.

Besides, from the average time data in Table 2, we can see that the IUPF calculation time of a single Monte Carlo is significantly shorter than the UPF and EKF-PF, and takes about half the amount of time. This is because the number of sampling points is significantly reduced by IUPF, making the calculation complexity become almost half of the original.Compared with PF, IUPF has higher tracking accuracy. This is because the IUPF method is used to generate the importance proposal distribution, so much more accurate mean and covariance can be updated. Compared with the UPF, IUPF can significantly reduce the calculation time while ensuring the positioning accuracy and has certain engineering application value. Therefore, the algorithm in this paper effectively improves the calculation efficiency while ensuring the basic performance.

**Figure 9:**
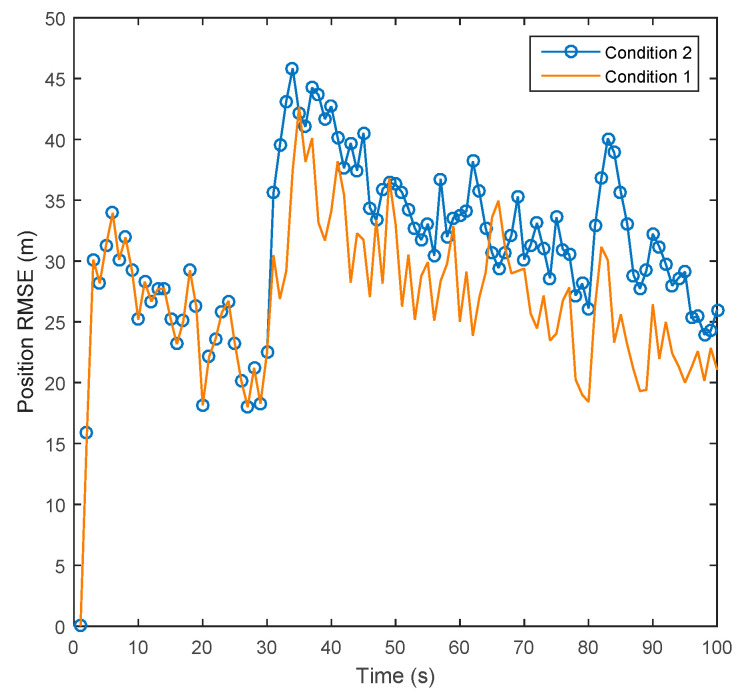
Comparison of position RMSE in adaptive gain correction experiment.

To prove whether the adaptive gain correction is effective for improving tracking accuracy in the IUPF algorithm, the other two experiments were carried out. One considers the gain correction coefficient and the other does not. Then, we extract target position state estimates at the 10th, 40th, 70th, and 90th seconds from the five stages of target motion and calculate their RMSE. Finally, we compare the performance of the above two experiments, whose results are shown in Figure 9 and Table 3.

Conditions 1 and 2 represent the added filtering gain correction in (17) to (19) and the unadded gain correction, respectively. From Figure 9 and Table 3, we can find that the adaptive gain correction is highly effective. Comparing Conditions 1 and 2, under the condition of strong maneuverings, such as the 40th and 70th seconds, for Condition 1, the adaptive gain correction coefficients are 0.6285 and 0.5621, respectively. Gain correction keeps the tracking error of the maneuvering target within a small range; for Condition 2, the error of the target state estimation increases significantly when the target is maneuvering and continues to affect the tracking accuracy of subsequent moments.

### 6.2. Multi-Target Tracking Simulation Analysis

**Figure 10:**
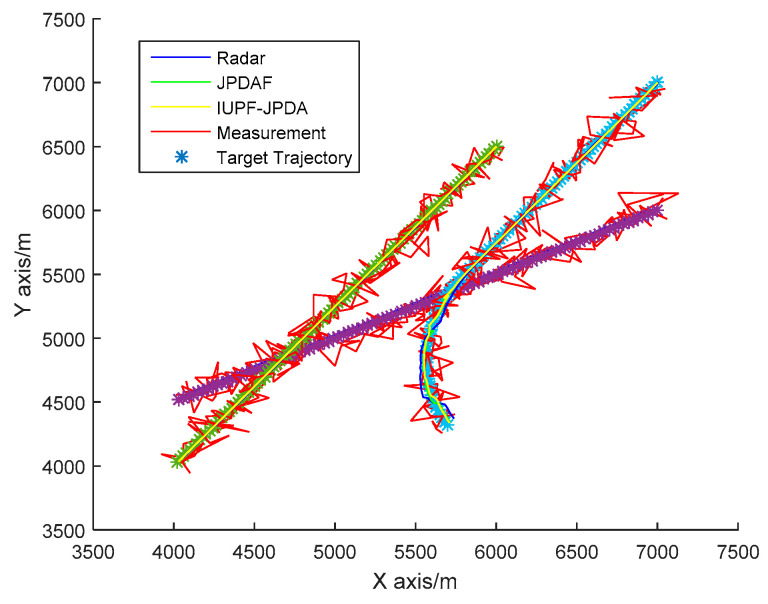
Multi-target simulation experiment prediction trajectories.

**Figure 11:**
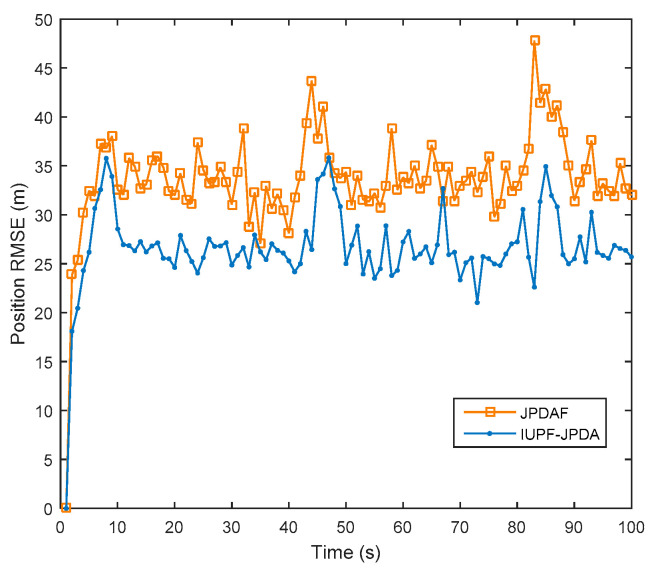
Comparison of position RMSE for target 1 in a multi-target simulation experiment.

**Figure 12:**
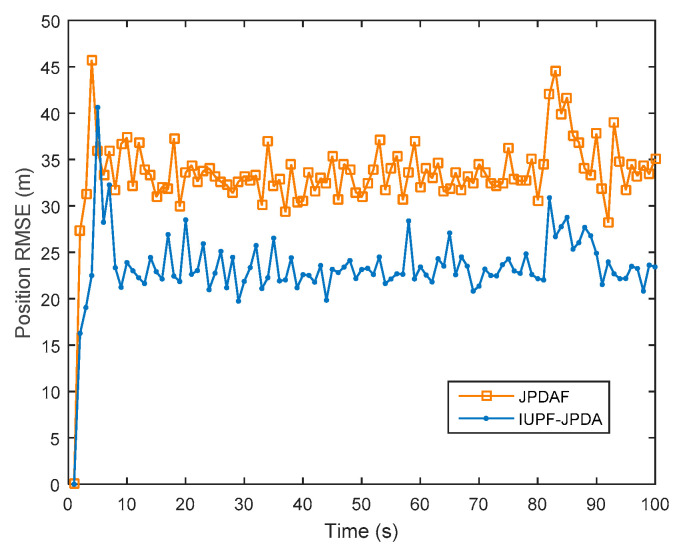
Comparison of position RMSE for target 2 in a multi-target simulation experiment.

**Figure 13:**
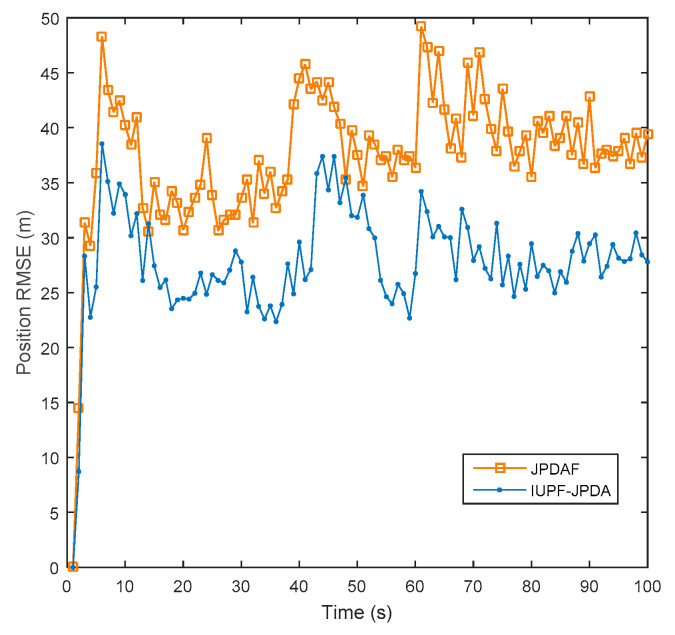
Comparison of position RMSE for target 3 in a multi-target simulation experiment.

Data association is key to enable true multi-target tracking. To verify the effectiveness of the algorithm proposed in Section 4, we compare the JPDAF algorithm with the IUPF combined with the JPDA algorithm. Figure 10 shows the comparison of prediction trajectories for three targets. Figure 11, Figure 12 and Figure 13 show the position errors of the three targets, respectively. Table 4 shows the average tracking error of different data association algorithm for multiple targets.

From Figure 11, Figure 12 and Figure 13, we can also find that when the trajectories of multiple targets cross and overlap, the position error will increase greatly. For example, the motion trajectories of target 1 and target 3 partially overlap at the 40th second, then the motion errors of the two targets both increase, but the multi-sensor fusion has lower fluctuations than the single sensor. Furthermore, we compare the traditional distributed fusion model with the improved model in this paper and find that the improved model has a smaller tracking error, higher accuracy, and better overall convergence effect.

For multi-target tracking, the data association algorithm is very important because it can handle the association between the target and the measurement. From Table 4, we can see that combining the IUPF and JPDA algorithms provides good performance for multi-target tracking. This is because the IUPF algorithm provides a more accurate prior probability distribution for the JPDA algorithm, and therefore the IUPF algorithm can get higher estimation accuracy.

## 7. Conclusions

In this paper, the multi-sensor fusion with IUPF and a new multi-sensor fusion model were designed, and we combine the IUPF algorithm with the JPDA algorithm for multi-target tracking. According to simulations, our conclusions are as follows.

- In this paper, based on the advantages and disadvantages of radar and infrared sensors, an improved fusion architecture is proposed based on the traditional distributed fusion architecture. Simulation experiments show that multi-sensor fusion can perceive increasingly accurate information compared with single sensors, and greatly improves the tracking accuracy, while the improved distributed multi-sensor fusion architecture can make better use of the advantages and disadvantages of radar and infrared sensors.
- The IUPF algorithm greatly improves real-time performance while ensuring tracking accuracy, which is undoubtedly a good method for tracking systems with real-time requirements.
- The proposed multi-target tracking method is a simple JPDA algorithm with IUPF, which has good performance.

Because the drastic reduction in the number of sample points will inevitably result in particle starvation, the particle filter resampling steps should be optimized at the foundation of existing theories. Besides, fuzzy logic, neural network, and wavelet transform should be integrated into the fusion algorithm of track transformation.

## Figures and Tables

**Table 1:** Comparison of average data for fusion models.

Algorithm	Radar	Traditional Model	Improved Model
**Average RMSE (m)**	57.351	32.463	25.945

**Table 2:** Comparison of average data in a single-target simulation experiment.

Algorithm	Average Time (s)	Average RMSE (m)
EKF-PF	0.602	39.316
PF	0.413	53.623
UPF	1.279	33.134
IUPF	0.68	26.214

**Table 3:** The effect of adaptive gain correction on target tracking accuracy.

	Time	10 s	40 s	70 s	90 s
**Condition 1**	X-RMSE (m)	27.56	34.21	29.16	28.65
	Y-RMSE (m)	31.22	36.12	26.36	27.41
	$\theta_{i,k}$	1	0.6234	0.5621	1
	X-RMSE (m)	27.56	35.21	32.67	31.98
**Condition 2**	Y-RMSE (m)	31.22	39.31	33.54	32.13

**Table 4:** Comparison of average data in a multi-target simulation experiment.

	Algorithm	Radar	JPDAF	IUPF-JPDA
Average RMSE (m)				
Target1		60.35	25.46	19.54
Target2		55.43	21.57	17.98
Target3		76.54	36.53	29.76
